# Determination of Conrad and Curie point depth relationship with the variations in lithospheric structure, geothermal gradient and heat flow beneath the central main Ethiopian rift

**DOI:** 10.1016/j.heliyon.2022.e11735

**Published:** 2022-11-17

**Authors:** Muluken Kassa, Abera Alemu, Ameha Muluneh

**Affiliations:** a College of Natural and Computational Sciences, Debre Tabor University, P.O. Box 272, Debre Tabor, Ethiopia; b School of Earth Sciences, Addis Ababa University, P.O. Box 1176, Addis Ababa, Ethiopia

**Keywords:** Curie point depth, Moho depth, Conrad depth, Lithospheric mantle thickness, Heat flow, Spectral analysis

## Abstract

Spectral analysis of the pole reduced magnetic anomaly data and inversion of complete Bouguer anomaly data are employed here as there is no previous published data regarding for the determination of the Curie point depth (CPD), Conrad depth (CD) and lithospheric mantle thickness in the central main Ethiopian rift (CMER) and its environs. The results confirm that the CPD, range between 7.68 and 20.3 km, CD, range between 16 and 25 km and lithospheric mantle thickness, range between 13.4 and 27. 8 km. These results indicate that the CMER magnetic crust occur close to the CD and lithospheric mantle thickness, but below the Moho depth beneath the study area. Based on the results on CPD, we estimate the magnitude of the geothermal gradient and heat flow in the study area. The results confirm that the geothermal gradient, range between 32.4 and 65 °C km^−1^ and heat flow, range between 80 and 160 mWm^-2^. These results are found to be inversely correlated with the CPD. It is a commonly known fact that shallow CPDs generate negative magnetization. Similarly, in this study, it is recorded low magnetic anomalies overlap with shallow (less than 13.1 km) CPDs in line with high (110–160 mWm^−2^) heat flow and high (48–64 °C km^−1^) geothermal gradient values are determined to occur beneath the CMER. These results associate with the presented geotectonic and geothermal signatures of the study area.

## 1 Introduction

The Curie point depth (CPD) is the theoretical surface thought to have been formed by ferromagnetic minerals in crustal rocks becoming paramagnetic assumed to have a temperature of ∼580 °C above which ferromagnetic minerals loss their magnetism and considered to be an indicator of the bottom interfaces of magnetized bodies (Wen et al., 2019).

Detailed understanding of the CPD is essential to reflect the deep structures of a magnetic body and to the understanding of the regional geological tectonics and evolution of a region. Understanding the CPD is also essential to provide vital information for the assessment of geothermal deposits, hydrocarbons deposits and mineral resources.

CPD calculation is performed based on the techniques used in magnetic and geothermal methods. These methods have their own contributions and limitations (Pilchin and Eppelbaum, 1997; Eppelbaum and Pilchin, 2006; Selim and Aboud, 2013). Pilchin and Eppelbaum (1997) suggested that geothermal methods are effectively applied in areas of geosynclines and stable platforms. However, they are not applicable at great depths (Eppelbaum and Pilchin, 2006). Unlike the geothermal methods, magnetic methods are based on utilization of magnetic data to calculate the CPD on a regional scale (Selim and Aboud, 2013). Multiple investigations (e.g., Espinosa-Cardeña and Campos-Enriquez, 2008; Obaje, 2009; Eletta and Udensi 2012; Salako 2012; Abdulsalam et al., 2013; Ikumbur et al., 2013; Igwesi and Umego 2013; Saibi et al., 2015; Bello et al., 2017; Mohammed et al., 2019; Abdullahi et al., 2019; Elbarbary et al., 2022) utilized aeromagnetic magnetic data elsewhere in order to determine the CPD on both a regional and local scale.

The Ethiopian Rift (ER) including the Main Ethiopian rift (MER) and Afar rift has a high geothermal power potential. In order to locate the areas that contain a high geothermal power potential beneath the region, the CPD, geothermal gradient and heat flow variations are determined. Several previous studies have conducted geophysical surveys in order to study geothermal resources (e.g., Endeshaw, 1988; Samrock et al., 2015, 2018, 2020; Cherkose et al., 2018; Cherkose and Saibi, 2021; Dejene et al., 2021), subsurface temperature distributions (e.g., Gianelli and Teklemariam, 1993; Teclu, 2002), geothermal gradient (e.g., Abiye, 2003), depth to Moho and lithosphere-asthenosphere boundary (LAB) (Kassa et al., 2021, 2022) and crustal wave velocity ratios (e.g., Dugda et al., 2005; Stuart et al., 2006; Keranen et al., 2009; Cornwell et al., 2010) beneath the ER and its environs. Cherkose and Saibi (2021) conducted magnetotelluric (MT) survey beneath the Ayrobera geothermal field within the Afar rift to characterize the subsurface as electrically conductive and resistive geologic horizons. They found shallow (down to a depth of 1.5 km) and deep (below 8 km) electrical conductive geologic horizons and high resistive geologic horizon appear to be in between the above mentioned shallow and deep electrical conductive zones (or a depth down from 1.5 to 8 km). According to different literature (e.g., Wannamaker et al., 2004; Arnason et al., 2010), the high electrical resistive zone is related with geothermal reservoirs comprising hot fluids within fractured rocks. This phenomenon is also observed in the Tulu-Moye and Aluto geothermal regions (e.g., Samrock et al., 2015, 2018, 2021; Cherkose and Mizunaga, 2018; Cherkose et al., 2018). Samrock et al. (2018) observed that the occurrence of upper crustal deformation at greater depth of approximately 8 km beneath the Tulu-Moye geothermal area. Kassa et al. (2021, 2022) conducted gravity studies using global gravity model plus (GGMplus 2013) acceleration data to determine gravity Moho, isostatic Moho, LAB depth and vertical stress beneath the main Ethiopian rift including adjoining plateaus. Here, we use ground magnetic data to calculate the CPD and GGMplus2013 acceleration data to determine the bottom interface of the lower crystalline basement (referred to here as “Conrad depth”) depth. Spectral analysis of magnetic data and GMSYS 3D inversion (Popowski et al., 2006) of gravity data is employed here as there is no previous published data regarding for the determination of the CPD and Conrad depth in the central main Ethiopian rift (CMER). Finally, we estimate the variations of geothermal gradient and heat flow from the CPD variations (e.g., Maden, 2010; Kasidi and Nur, 2012) and discuss the CPD results in relation with geothermal gradient variation, heat flow variation, crustal wave velocity ratio anomalies (e.g., Wen et al., 2019), lithospheric structures including Moho and Conrad depths.

## 2 Geological and structural setting

**Figure 1:**
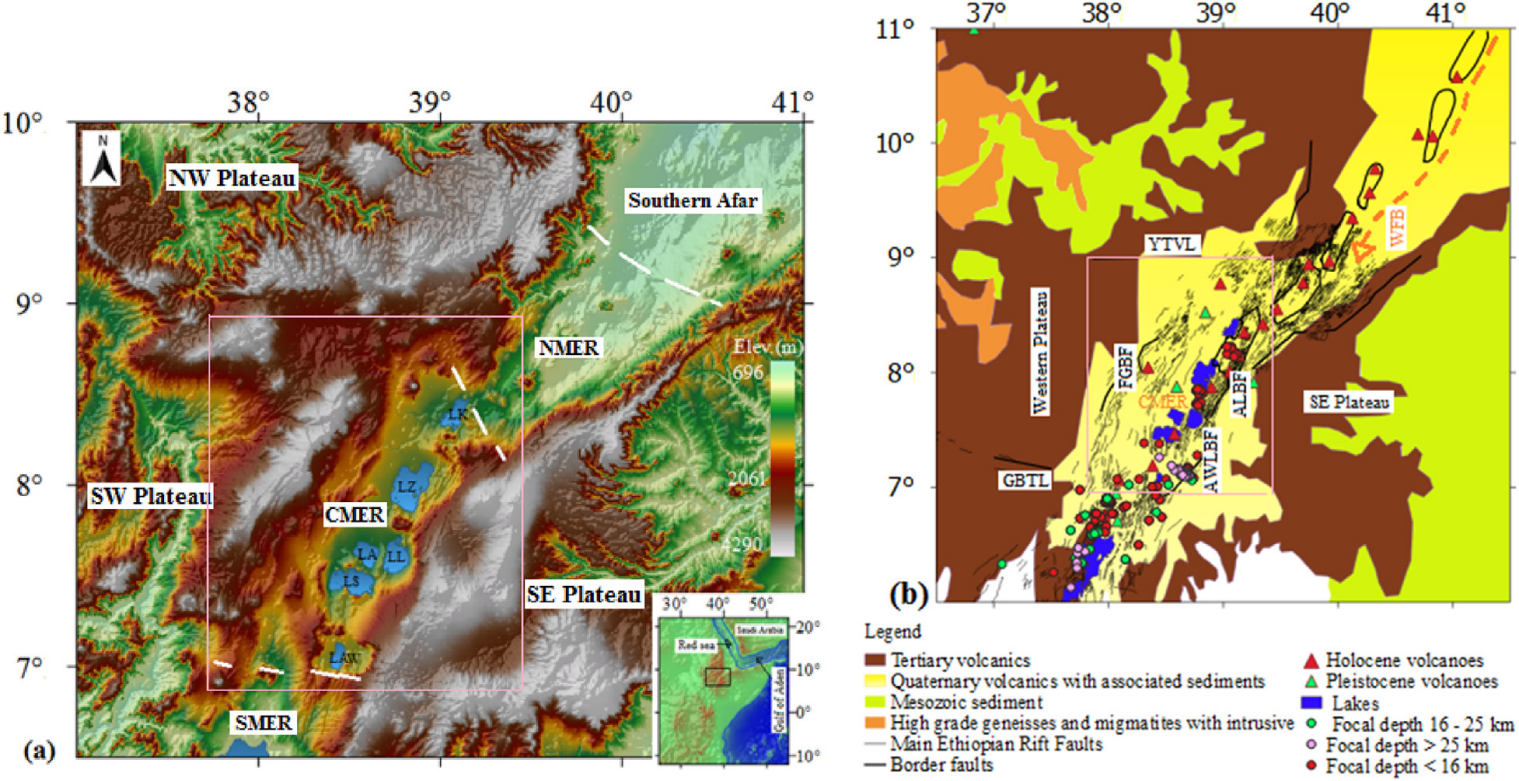
(a) Location map of the main Ethiopian rift (MER) comprising the surrounding regions (i.e., the southwestern (SW), northwestern (NW) and southeastern (SE) plateaus). The MER Physiographically can be grouped into the northern main Ethiopian rift (NMER), central main Ethiopian rift (CMER) and southern main Ethiopian rift (SMER). The central MER consists of different lakes including the Lake Koka (LK), Lake Ziway (LZ), Lake Abiyata (LA), Lake Langano (LL), Lake Shalla (LS) and Lake Awassa (LAW). (b) Geological map of the central MER and surrounding regions (Tefera et al., 1990). The CMER is surrounded by the Fonko-Guraghe border fault (FGBF) to the west, Yerer-Tullu Wellel volcanotectonic lineament (YTVL) to the northwest, Awassa-Langano border fault (AWLBF) to the southeast, Asela-Langano border fault (ALBF) to the northeast and Goba-Bonga tectonic lineament (GBTL) to the south. Black lines and polygons represent the major faults (e.g., Agostini et al., 2011) and Wonji fault belt (WFB), respectively. White dashed lines outline the boundaries of the northern, central and southern MER (e.g., Corti, 2009). Pink rectangles inside a, b, shows the area of interest.

The study area, which is the CMER (Figure 1a, b, open pink rectangular shape), is flanked by the Fonko-Guraghe fault escarpment (FGFE) to the west and the Awassa-Langano (AWL) including the Asela-Langano (AL) fault escarpments (FE) to the east (Figure 1a, b). It is also bounded by the E-W oriented Yerer-Tullu Wellel volcanotectonic lineament (YTVL) to the north and Goba-Bonga tectonic lineament (GBTL) to the south. The CMER is influenced by the NNE-SSW trending Wonji-fault belt (WFB) and the Oligocene-Miocene border faults (Figure 1b) that separate the rift from neighboring western (i.e., northwestern and southwestern plateaus) and southeastern (SE) plateaus. The rift floor and surrounding plateaus are covered by volcanic rocks (Kazmin, 1975, Figure 1b). In the study area, most of the Holocene and Pleistocene volcanoes are distributed along the rift floor (Figure 1b), acquired from the Global volcanic program (https://volcano.si.edu). In the study area, major faults mainly occur in the western and eastern margins of the rift with minor activity in the rift floor (Agostini et al., 2011; Corti et al., 2020).

Clear crustal thickness contrasts occur beneath the CMER and surrounding plateaus (e.g., Keranen et al., 2009; Kassa et al., 2021). Beneath the central MER, the crustal thickness varies from 38 to ∼45 km (Kassa et al., 2021) which includes an average upper crustal thickness of ∼20 km (Keranen et al., 2009). Keranen et al. (2009) suggested that the lower crust beneath the CMER is characterized by basaltic composition. The composition of the upper crust beneath the CMER and southeastern plateau is thought to consist of feldspar and silicon (Keranen et al., 2009; Mackenzie et al., 2005). Numerical modelling results by Muluneh et al. (2020) suggest that the whole crust in the MER is characterized by wet quartzite composition.

Previous seismic studies (e.g., Keir et al., 2006; Wilks et al., 2017; Greenfield et al., 2019; Lavayssiére et al., 2019; Lapins et al., 2020) which this study has made use of indicate that earthquakes with focal depths ranging between 0.1 and ∼39 km occurred beneath the central MER and eastern margin of the rift (Figure 1b).

## 3 Methods

### 3.1 Spectral analysis technique

Spectral analysis technique were applied to magnetic data to estimate the CPD in different parts of the world comprising Greece (Tselentis, 1991), the Kamchatka Peninsula (Tanaka, 2007), Mexico (Manea and Constantin, 2011), Red Sea (Abd El Nabi, 2012), central India (Bansal and Anand, 2013) and Egypt (Ahmad, 2019). Here, we employ the spectral analysis technique in order to calculate the top, centroid and bottom depths of magnetized bodies. According to Nwankwo and Shehu (2015), calculations of top and centroid depths of magnetized bodies are dependent on the highest and lowest wavenumber parts of the power spectrum, respectively. Centroid (*Z*_*o*_) and top (*Z*_*t*_) depths are given by Eq. (1) and Eq. (2), respectively.

(1) ln(P(k)12|k|)=A−|k|Zo,

Where *P*(*k*)is power density spectrum, *k* is wavenumber and *A* is a constant.

(2) ln(P(k)12)=B−|k|Zt,

Where *B* is a constant.

Calculation of CPD (*Z*_*b*_) is dependent on centroid (*z*_0_) and top (*z*_*t*_) depths of the magnetized bodies (e.g., Maden, 2010) and is given by Eq. (3).

(3) $$Z_{b}=2Z_{0}-Z_{t}.\;$$

### 3.2 Conrad depth inversion methodology

We use GMSYS 3D inversion (Popowski et al., 2006) to generate the depth to Conrad beneath the central MER and surrounding region. GMSYS 3D inversion uses the algorithm developed by Parker-Oldenburg (1973, 1974). The inversion algorithm is designed to run inside Geosoft Oasis Montaj software (Geosoftw program 2007) utilized to process and analyze gravity data. The Parker-Oldenburg inversion algorithm were used successfully by various authors (Tugume et al., 2013; Dogru et al., 2018; Kassa et al., 2021) to determine the depth to Moho, basement and LAB depth in the areas where they studied. The GMSYS 3D inversion iterates the Fourier analysis of the gravity anomaly, and inverts it based on the Conrad reference depth through the inverse Fourier transform. In this inversion, we consider the depth to Conrad to be represented by the bottom density interface of the lower crystalline basement. Among other functionalities, the GMSYS 3D inversion performs Conrad depth inversion modelling as a function of reference depth to Conrad, determines density contrast values for the interface occurring between the upper and lower crust, and filtering cutoff wavelength. In applying the GMSYS 3D inversion to the CMER and surrounding plateaus, we assigned an average depth to Conrad has a value of 20 km acquired from the earlier wide angle controlled source seismic imaging (e.g., Maguire et al., 2006) and a density contrast value of 150 kg/m^3^ for the interface occurring between the upper and lower crust (Maguire et al., 2006).

Calculation of density contrast value can be expressed using Eq. (4) as follows:

(4) DensitycontrastvalueattheConrad=Lowercrustdensity−Uppercrustdensity

Where the lower crust density value is amounts to 2.94 g/cc and upper crust density value is equal to 2.79 g/cc beneath the study area (Mackenzie et al., 2005; Maguire et al., 2006; Keranen et al., 2009). These density values are estimated from the available p-wave velocity model (e.g., Maguire et al., 2006) by using p-wave velocity -density conversion of Nafe and Drake (1957) given by Eq. (5).

(5) $$\rho =-168.03+1765*V_{p}-481.72*V_{p}^{2}+60.973*V_{p}^{3}-2.6861*V_{p}^{4}\;,$$

where *ρ* is the density of the model layers used and *V*_*p*_ is the P-wave velocity (in km/s).

### 3.3 Magnetic and gravity data processing and analysis

#### 3.3.1 Total magnetic intensity (TMI) field

Many scholars have used aeromagnetic data (e.g., Tselentis, 1991), world digital magnetic anomaly map data (e.g., Maus et al., 2007), Earth Magnetic Anomaly (EMAG2) data (e.g., Nwankwo and Shehu, 2015; Ahmad, 2019; Maden et al., 2020) and ground magnetic data (Selim and Aboud, 2013) to estimate the CPD in the regions where they studied.

**Figure 2:**
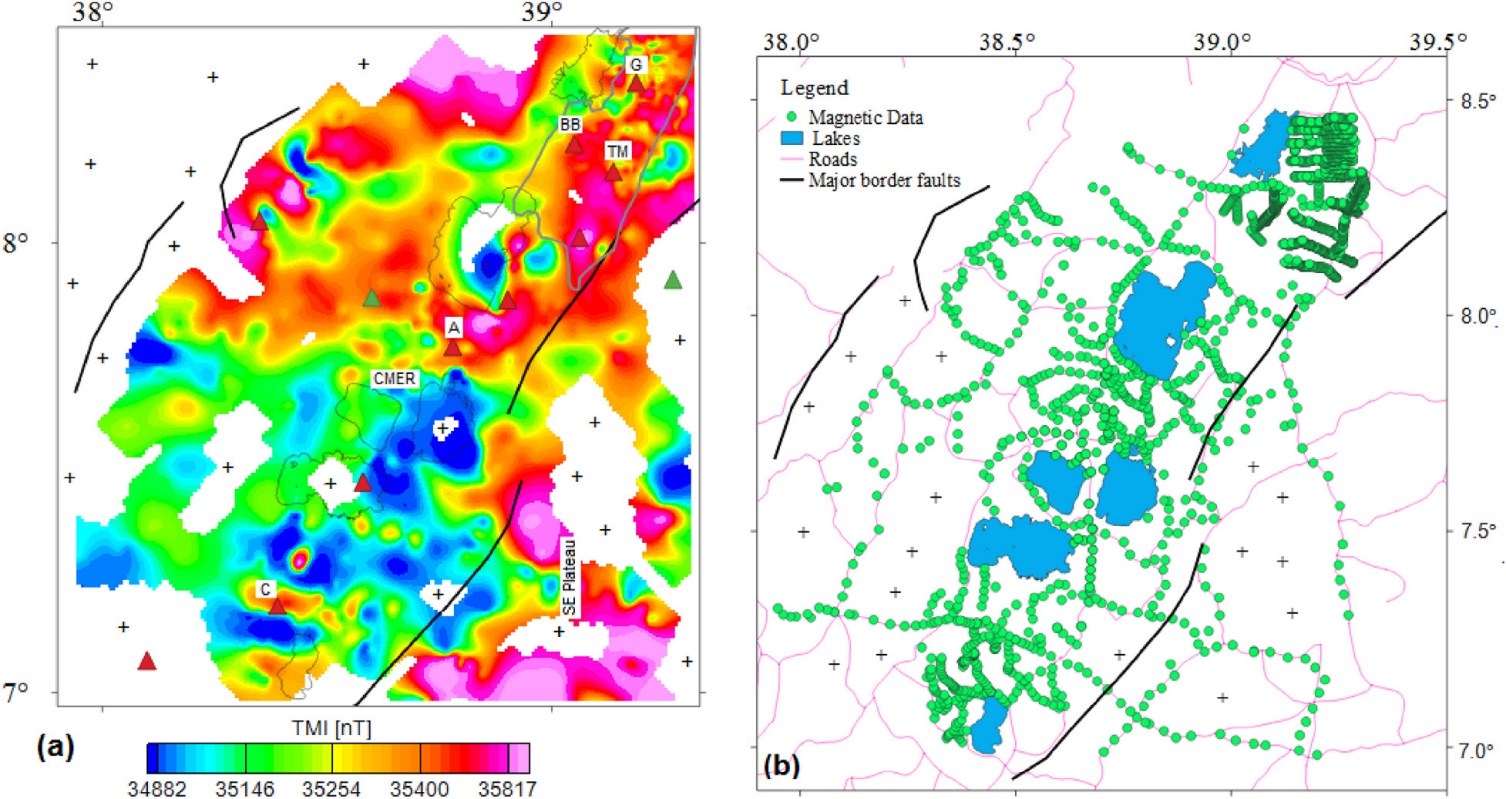
(a) Total Magnetic Intensity field map of the Central Main Ethiopian Rift (CMER) and the southeastern plateau (SE Plateau). (b) Distributions of the magnetic data in the CMER and SE Plateau. Plus signs represent the magnetic data gaps. G, BB, TM, A and C stands for the Gedemsa, Bora-Bericcio, Tulu-Moye, Aluto and Correbetti volcanic complex, respectively.

A total of about 2335 secondary including primary total magnetic intensity (TMI) data are used to compute the CPD beneath the central MER and surrounding regions. The secondary TMI data were obtained from MSc works of numerous scholars (Kebede, 2014; Berhane 2015; Kelemework, 2016; Nigussie, 2020) and Kebede et al. (2021) guided by their advisor Dr. Abera Alemu. The Author and his advisor (Dr. Abera Alemu) have collected more than 200 primary TMI data using proton magnetometer. The secondary and primary TMI data distributions are shown in Figure 2b, which are gridded and mapped using Geosoft Oasis Montaj (Version 7.0.1) software to generate total magnetic anomaly (TMA) and its derivative map (Sections 3.4.2.1).

#### 3.3.2 Total field magnetic anomaly

**Figure 3:**
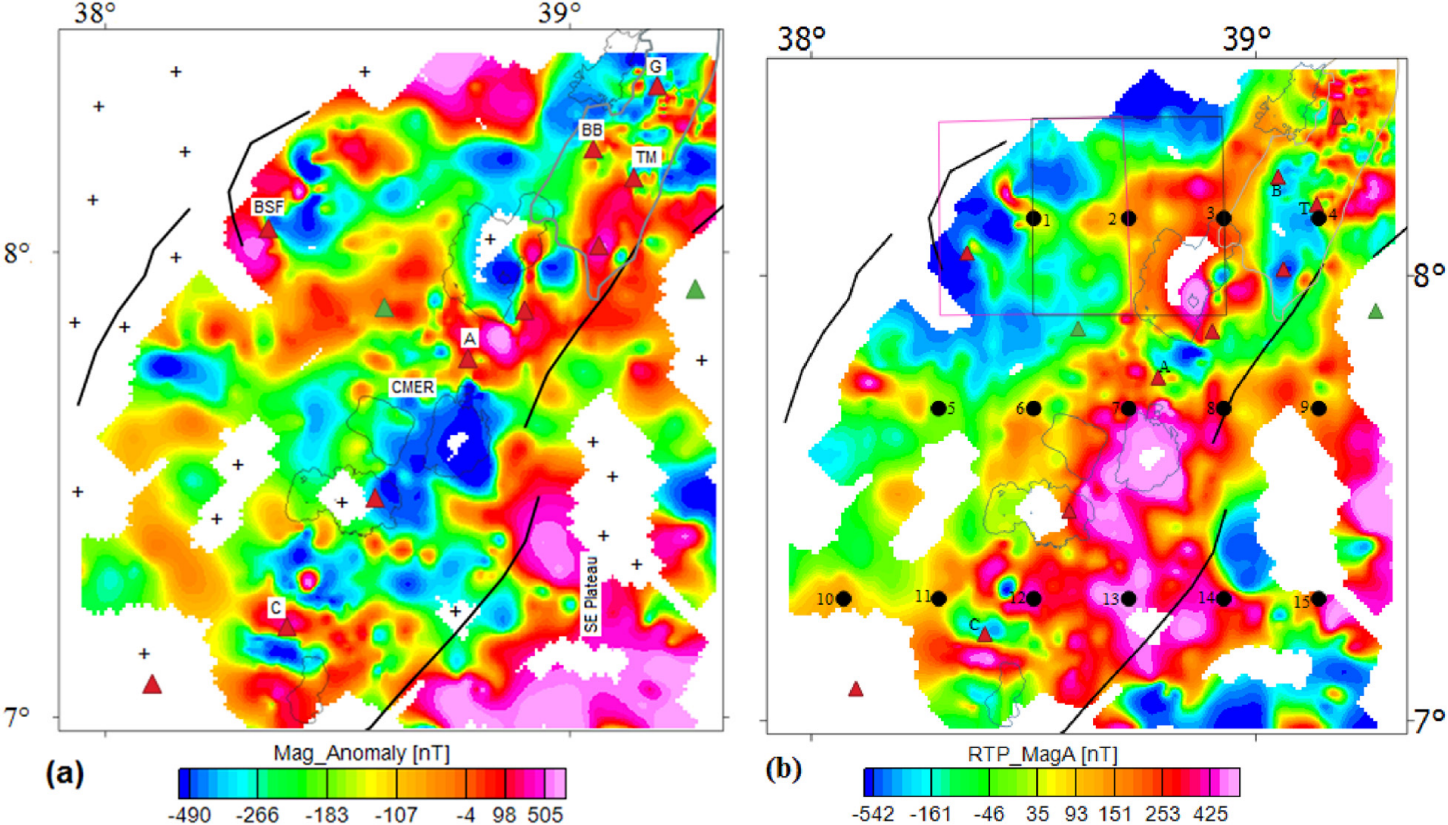
Total field magnetic anomaly map (a) and RTP magnetic anomaly map (b) of the study area. Pink and black rectangles represent selection of overlapping windows on the RTP magnetic anomaly map. Black dots indicate centers of windows. Plus symbols on the TMA map represent the lack of magnetic data. G, BB, TM, A and C stands for the Gedemsa, Bora-Bericcio, Tulu-Moye, Aluto and Correbetti volcanic complex, respectively. The acronym BSF stands for the Butajira-Silti field.

The TMA (Figure 3a) is computed by subtracting the theoretical main field (IGRF, 2005 field) from the TMI field (Figure 2a). In Figure 3a, blue colored zones are represented by minimum magnetic amplitude and red to pink colored zones are represented by maximum magnetic amplitude, which are thought to indicate high and low magnetic anomaly zones, respectively. It is recognized that interpretation of the TMA is complicated because of the proximity of the study area to the magnetic equator. The utilized magnetic data in this work is collected near to the magnetic equator (declination = 1.95°; inclination = −0.12°), interpretation is difficult for several grounds including the ambient geomagnetic field is weak and it is oriented horizontally; and N–S features are magnetically hidden. To avoid this inherent complication in interpretation, the reduction to the pole (RTP) filter is applied to the gridded TMA so as to generate the RTP map (Figure 3b) derived from the TMA. However, it is recognized that the RTP filters are unstable in low latitude area (i.e., <30° inclination). This unsteadiness trouble is tackled in this work by applying strong amplitude corrections (in our case-uses an amplitude inclination of 39°) during pole reduced process.

##### 3.3.2.1 RTP magnetic anomaly

The compiled RTP magnetic anomaly map (Figure 3b) is compiled for the average inclination (−0.12°), declination (1.95°) and total intensity value (35507 nT) of the study area. Unlike the TMA map (Figure 3a), the blue and red to pink colored zones represent low and high magnetic anomaly areas, respectively (Figure 3b).

Comparing TMA map (Figure 3a) and RTP magnetic anomaly map (Figure 3b) beneath the investigated area don't show the same geological correlation. That is, Figure 3b reveals high magnetic anomalous zone, whereas Figure 3a shows low magnetic anomalous zone for the same locations beneath the central MER, except at the location of the Aluto volcanic complex where the Aluto volcanic complex is marked by a negative anomaly signature on the RTP magnetic anomaly map (Figure 3b). The high magnetic anomalies zones reveled by the RTP map (Figure 3b) beneath the central MER appear to be associated with basaltic minerals (Maguire et al., 2006). The negative anomaly signatures associated with the Aluto volcanic complex could be thought to be the effect of sediments filling the Aluto caldera. Hu (2009) suggested that tectonically active and stable areas are characterized by low and high magnetic anomalies, respectively. This scenario is very well depicted on the compiled RTP magnetic anomaly map (Figure 3b). The Correbetti volcanic complex is marked by negative RTP magnetic signatures indicates that the Correbetti volcanic complex is tectonically active area (Figure 3b).

### 3.4 Complete Bouguer gravity anomaly

**Figure 4:**
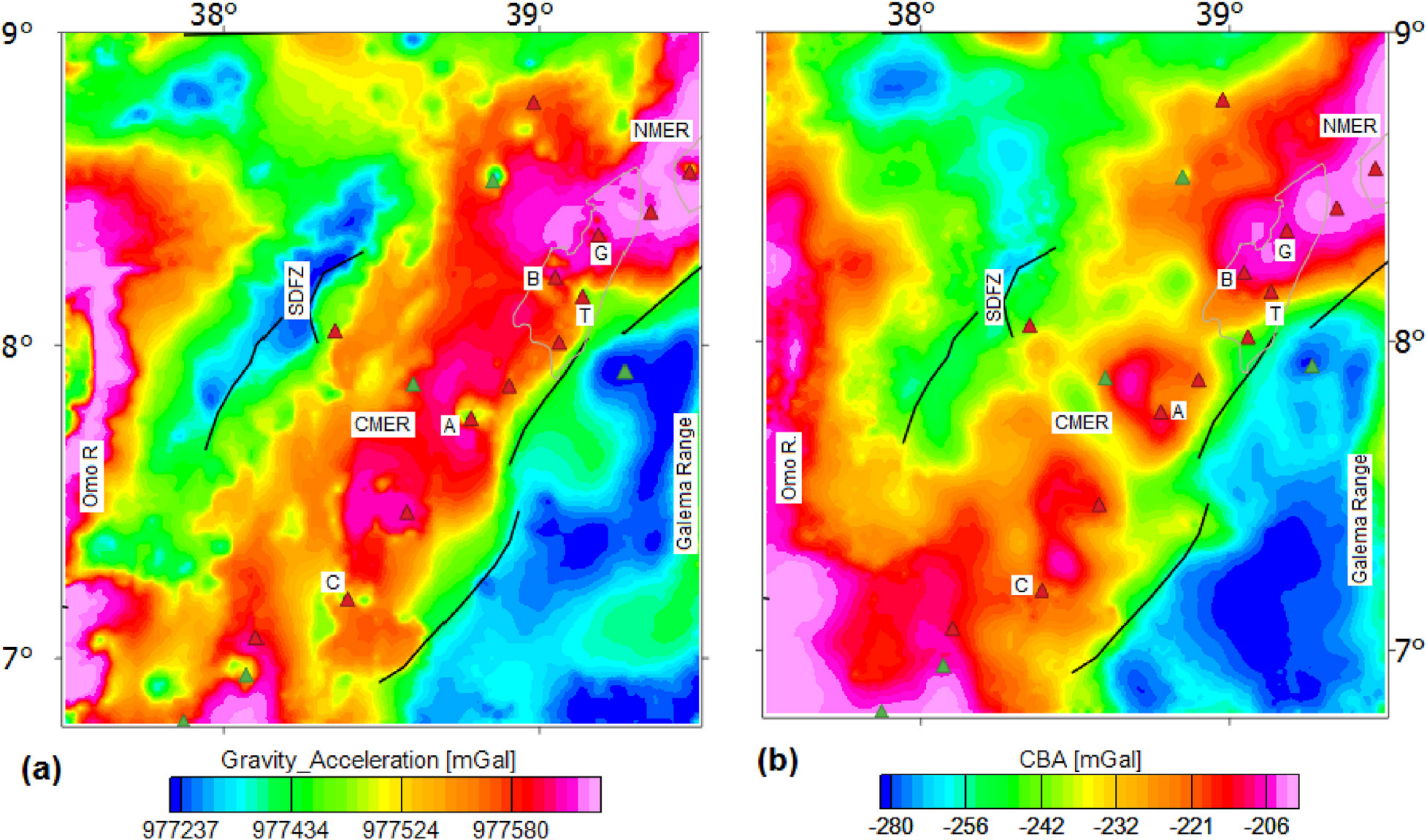
Gravity acceleration (a) and complete Bouguer anomaly (b) map of the study area. G, B, T, A and C stands for the Gedemsa, Bora-Bericcio, Tulu-Moye, Aluto and Correbetti volcanic complex, respectively. The acronyms include SDFZ, Omo R, NMER and CMER stands for the Silti Debrezit fault zone, Omo River, northern and central main Ethiopian rift, respectively.

The gridded gravity accelerations (Figure 4a) map of the region obtained from the Global Gravity Model plus (GGMplus 2013). The standard corrections are applied to the observed gravity acceleration values (Figure 4a) in order to compile the CBA for the study area considered. The compiled CBA map (Figure 4b) shows a relative Bouguer anomaly maximum (−164 mGal) beneath the rift and Omo river valley and relative minimum (−285 mGal) beneath the southwestern and southeastern plateaus. The anomaly maxima occurring over the CMER coincide with relatively thin (ranging from 38 to 40 km) crust (Chambers et al., 2019) and the anomaly minima occurring over the plateaus coincide with a relatively thick (51 km) crust (Mahatsente et al., 1999).

#### 3.4.1 Isolation of the CBA

**Figure 5:**
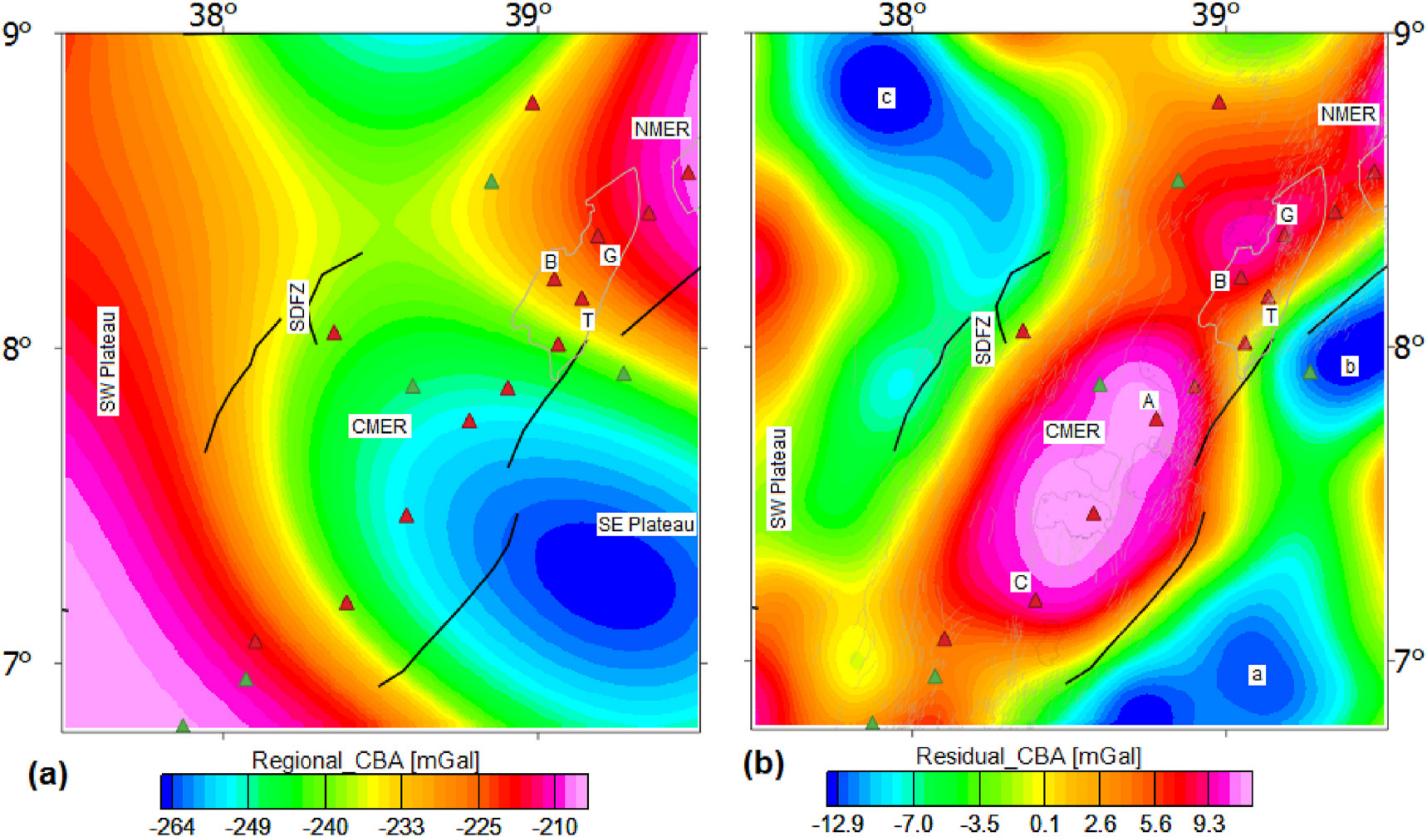
Regional (a) and residual (b) complete Bouguer anomalies map of the central MER and surrounding plateaus. G, B, T, A and C stands for the Gedemsa, Bora-Bericcio, Tulu-Moye, Aluto and Correbetti volcanic complex, respectively. The small letters a, b and c represent lower residual gravity anomaly areas. The acronyms include SDFZ, Omo R, SW plateau, NMER and CMER stands for the Silti Debrezit fault zone, Omo River, southwestern plateau, northern and central main Ethiopian rift, respectively.

The CBA is a combined effect of deep (regional) and shallow (residual) gravity sources. Several studies including our previous gravity studies (Salem et al., 2013; Kassa et al., 2021) have effectively used a low pass filter to separate the regional and residual gravity anomalies. Here, we employ a similar approach followed by Salem et al. (2013) and Kassa et al. (2021) to isolate the regional and residual gravity anomalies. The estimated regional and residual gravity anomalies are depicted in Figures 5a and b. The estimated residual gravity anomaly values (Figure 5b) fluctuate between −15.71 and 17.63 mGal beneath the study area. The residual gravity maxima appear to coincide with locations of the volcanic centers and magmatic segments occurring along the rift axis (Figure 5b). Sources of the residual anomalies are thought to have been distributed within the upper crust (Torne’ et al., 2015).

## 4 Results and discussion

### 4.1 Curie point depth

**Figure 6:**
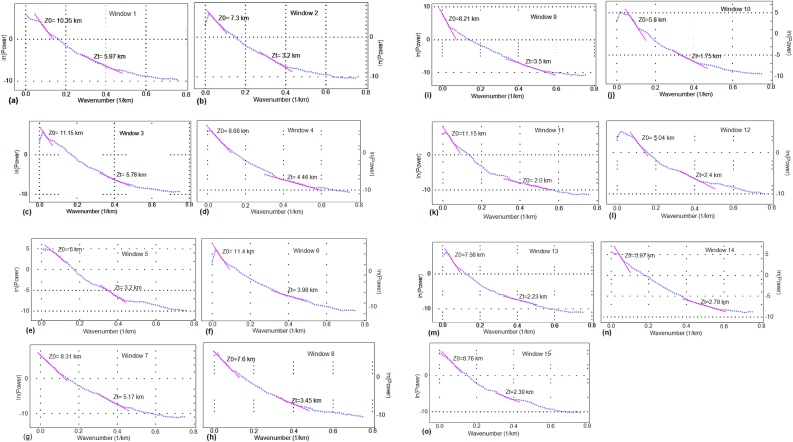
a–o Radially averaged power spectrum of the RTP magnetic anomaly for each window in the region.

Calculation of depths of magnetized bodies corresponding to centroid (*Z*_0_) and top (*Z*_*t*_) depths are presented in Figure 6a–o. These depths are utilized to model the CPD (Okubo et al., 1985; Maden, 2010) beneath the study area.

A number of studies (e.g., Maden, 2010; Okubo et al., 1985) have estimated the CPD elsewhere using centroid (*Z*_0_) and top (*Z*_*t*_) depths of magnetized bodies. These studies employed Fourier domain pole reduced magnetic anomaly data by dividing several overlapping windows (blocks) of different sizes. They also employed first order trend filter to remove the deep regional structures for each window (block) and the maximum entropy method to expand the grid considered by 10 % and to make the anomaly edges continuous.

A similar approach followed by Okubo et al. (1985) and Maden (2010) to estimate the CPD for the study area. Here, we used the Fourier domain pole reduced magnetic anomaly data by dividing the grid map into 15 overlapping windows of size 27.5 km × 27.5 km (overlapped 50 % with the adjacent windows).

Figure 6a–o shows the calculated centroid and top depths of the magnetized bodies within each window in the central MER and surrounding regions. The calculated top depth values range from 1.75 km to 5.97 km (Figure 6a–o), which is consistent with the earlier results of Maguire et al. (2006), who found the sedimentary-volcanic layer depths ranging from 2 to 5 km beneath the CMER. The estimated centroid depth values vary in the range of 5.04–11.4 km (Figure 6a–o), which is in line with the results of Maguire et al. (2006), who found the upper crystalline basement depth to vary in the range of 5–15 km beneath the CMER. Table 1 shows the estimated depths to the centroid, top and bottom magnetized bodies in the region.

**Table 1:** Estimated average Curie point depth, Depth to centroid and top interfaces from spectral analysis.

Windows no	Depth to centroid (km)	Depth to top interface (km)	Curie depth (km)
1	10.35	5.97	14.73
2	7.3	3.2	11.4
3	11.15	5.78	16.52
4	8.68	4.46	12.9
5	6.0	3.2	8.8
6	11.4	3.96	18.84
7	8.31	5.17	11.45
8	7.6	3.45	11.75
9	8.21	3.5	12.92
10	5.8	1.75	9.85
11	11.15	2.0	20.3
12	5.04	2.4	7.68
13	7.56	2.23	12.89
14	5.97	2.79	9.15
15	8.76	2.39	15.13

**Figure 7:**
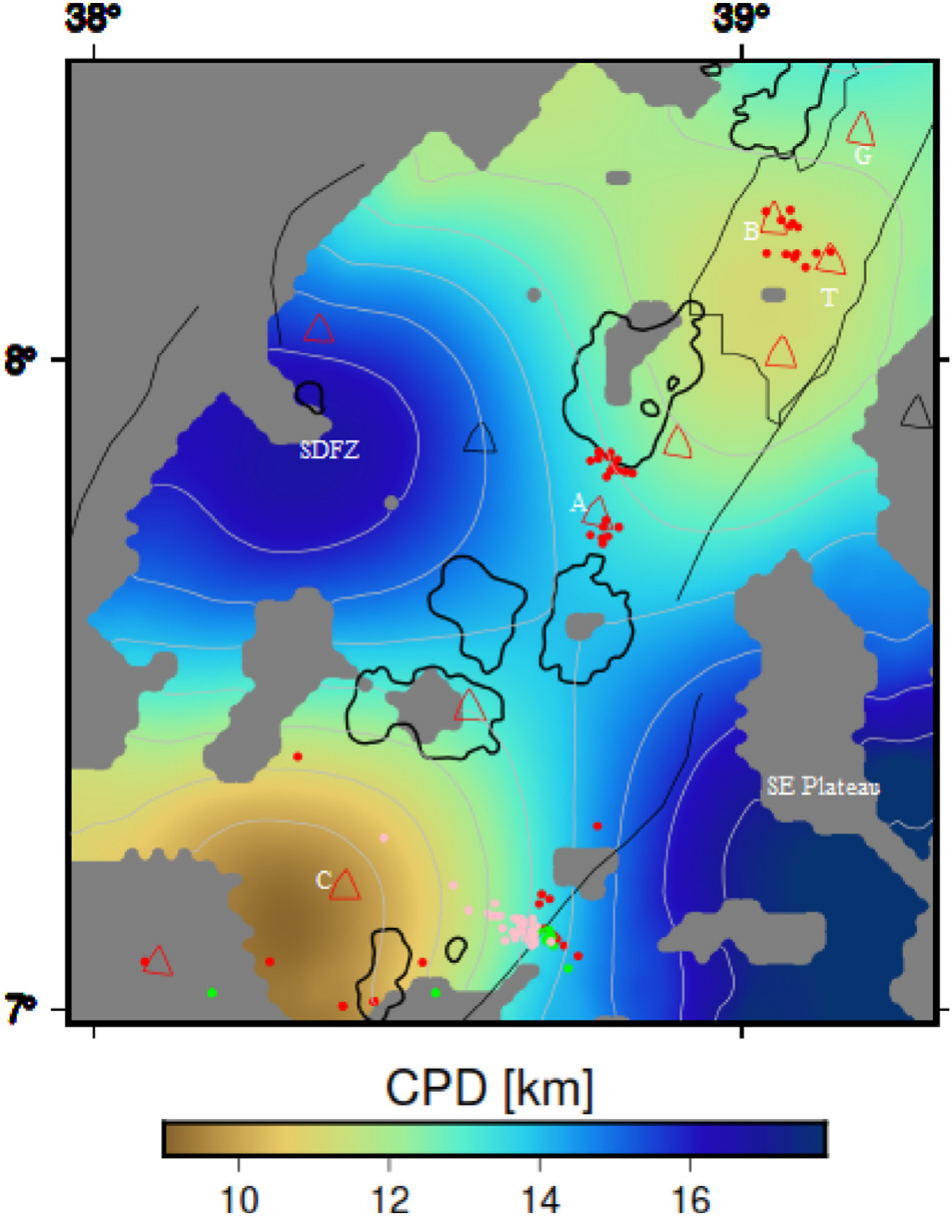
Curie point depth map of the investigated region, showing the shallow CPD coincide with volcanic (red and black circles), magmatic (gray polygon) and seismicity (red, green and pink circles) centers in the region. Black polygons represent the rift lakes. G, B, T, A and C stands for the Gedemssa, Bora-Bericcio, Tullu-Moye, Aluto and Correbetti central volcanoes. The acronyms include SDFZ and SE plateau stands for the Silti Debrezit fault zone and southeastern plateau, respectively.

The estimated depths to the centroid and top of the causative magnetic bodies are utilized for the determination of the CPD in the study area. The computed CPD map for the central MER and surrounding region is depicted in Figure (7). The estimated CPD for the volcanic complexes including the Gedemssa, Bora-Bericcio, Tulu-Moye, Aluto and Corbetti appear to be shallow varying between 7.68 and 13.1 km, which is in agreement with the result of Badalyan (2000), who found CPD values between 6 and 12 km beneath the thermally active area in Armenia. The shallow CPD determined for the volcanic complexes in the CMER is thought to be associated with geothermal sources and active volcanoes (Aydin and Oksum, 2010). The southeastern plateau and the SDFZ appeared to be characterized by high CPD values at a depth of 20.3 km (Figure 7).

Calculation of surface heat flow (*q*_*s*_) in terms of CPD (*Z*_*b*_) can be given using Eq. (4) (Li et al., 2013; Speranza et al., 2016; Wang and Li, 2015).

(6) qs=kTc−T0Zb−Z0+hr2H0e−Zbhr−e−Z0hrZb−Z0+hrH0e−Z0hr,

where k is thermal conductivity and taken here to be 2.5 W/m °C based on the average thermal conductivity of the crustal rocks (Stacey, 1977; Springer, 1999), Tc is the Curie temperature and considered here to be equal to 580 °C (Ross et al., 2006), Z0 is surface elevation and taken here to be 0, T0 is surface temperature and taken here to be 20 °C (Ayenew, 2002).

The rate of surface radioactive heat, H0, is considered here amounts to 2 μW/m^3^ and the thermal characteristic depth, hr, is equal to 10 km (Pasquale et al., 2014).

**Figure 8:**
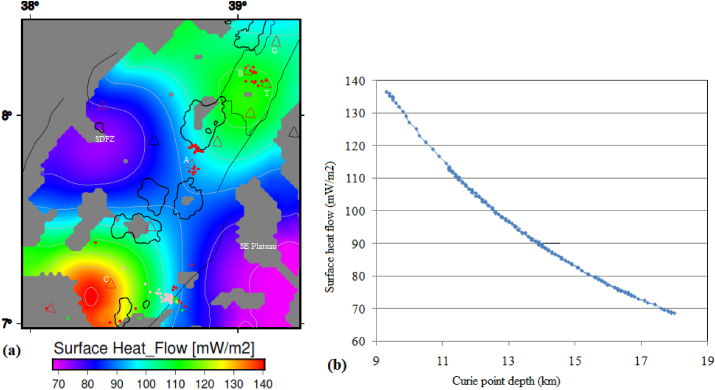
(a) Surface heat flow and (b) plot of the CPD versus surface heat flow of the study area. Black polygons represent the rift lakes. Red, green and pink circles represent the seismicity centers with depth less than 16 km, between 16 and 25 km and greater than 25 km, respectively. The texts include G, B, T, A and C stands for the Gedemssa, Bora-Bericcio, Tullu-Moye, Aluto and Correbetti central volcanoes. The acronyms include SDFZ and SE plateau stands for the Silti Debrezit fault zone and southeastern plateau, respectively.

The estimated surface heat flow (Fig. 8a) varies between 68.2 and 141 mW/m^2^ and the average surface heat flow is estimated to be ∼95 mW/m^2^. In most parts of the study area, the estimated surface heat flow values are above that of the previous results of Muluneh et al. (2021), who estimated a surface heat flow value of 70 mW/m^2^ for the MER incorporating the study area.

A plot of the estimated surface heat flow values against the CPD shows an inverse correlation for the study area (Fig. 8b). The highest surface heat flow values are observed to occur beneath the active volcanoes (geothermal zones) in the central MER (Fig. 8b). The active volcanic areas including the Corbetti, Tulu-Moye, Bora-Bericcio and Gedemssa appear to be associated with the highest surface heat flow values (141 mW/m^2^) corresponding to the lowest CPD values (up to 7.68–13.1 km) (Fig. 8b). The low surface heat flow values (68.2 mW/m^2^) appear to coincide with the deep (20.3 km) CPD values occurring in the southeastern plateau and the SDFZ (Fig. 8b). The observed inverse correlation between surface heat flow and CPD has been confirmed by several studies (Saada, 2016; Speranza et al., 2016) in the areas they studied.

Xu (2009) suggested that tectonically active and stable areas are characterized by low and high magnetic anomalies, respectively. Accordingly, the relatively deep CPD, high heat flow and low RTP magnetic anomaly values (Figure 3b, 7, 9a) observed beneath the Aluto volcanic complex appear to be correlated with active tectonics (Corti et al., 2020).

**Figure 9:**
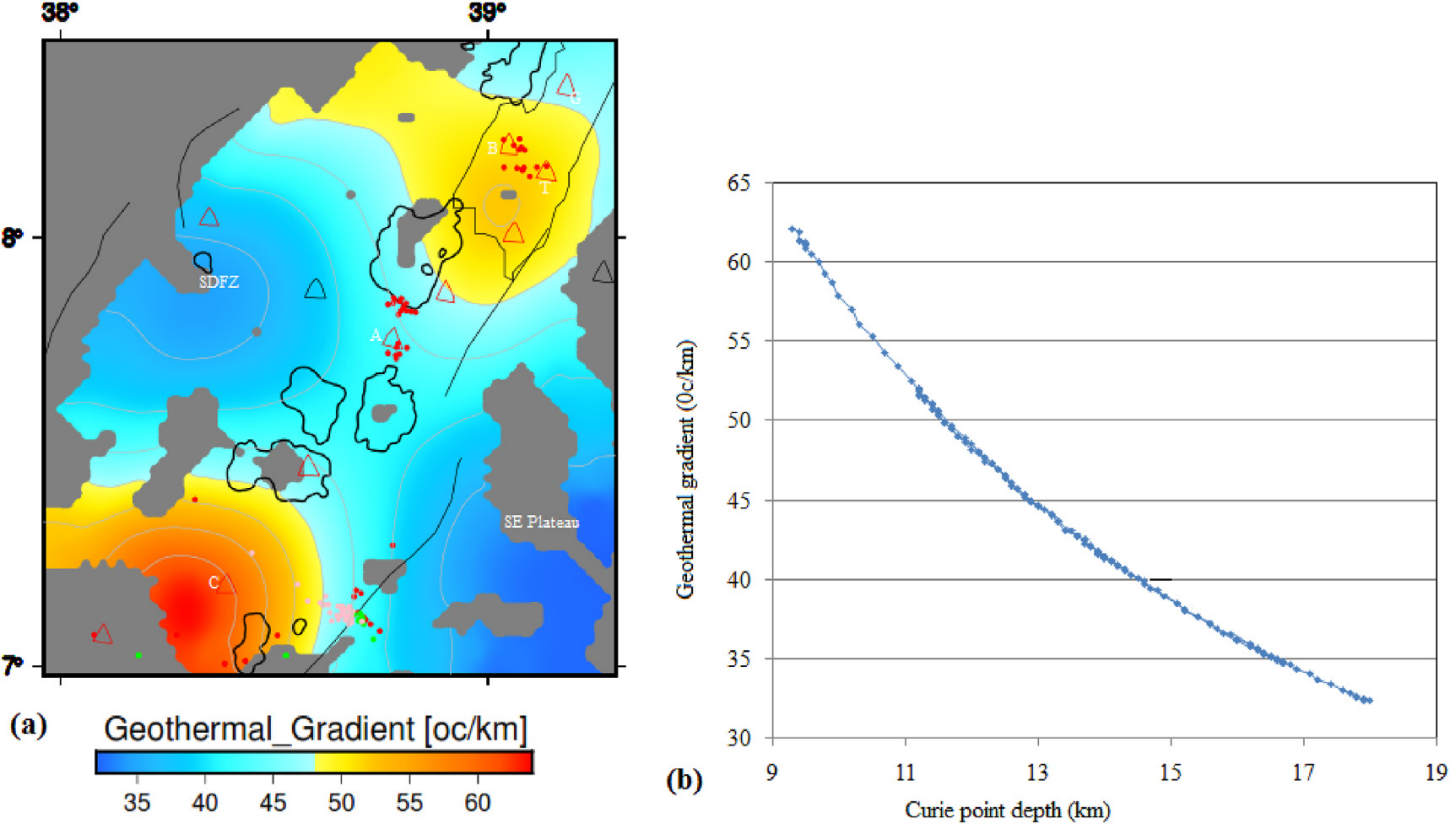
(a) Geothermal Gradient map and (b) plot of the CPD versus geothermal gradient of the region. Black solid line indicates the major boundary faults, black polygon represents lakes, grey polygon represent Koka magmatic segment and red triangle represents volcanic centers. SDFZ, G, B, T, A and C stands for the Silti Debrezit fault zone, Gedemssa, Bora-Bericcio, Tulu-Moye, Aluto and Corbetti, respectively.

#### 4.1.1 Comparison of CPD with geothermal gradient

The geothermal gradient of an area can be estimated using Eq. (5) (Tanaka et al., 1999; Stampolidis et al., 2005).

(7) $$\frac{dT}{dz}=\frac{T_{c}}{D},$$

where D is CPD; Tc is the Curie temperature and taken here to be 580 °C (e.g., Ross et al., 2006). This equation indicates that estimation of geothermal gradient depends mainly on the calculated CPD (Figure 8) map of any region.

**Figure 10:**
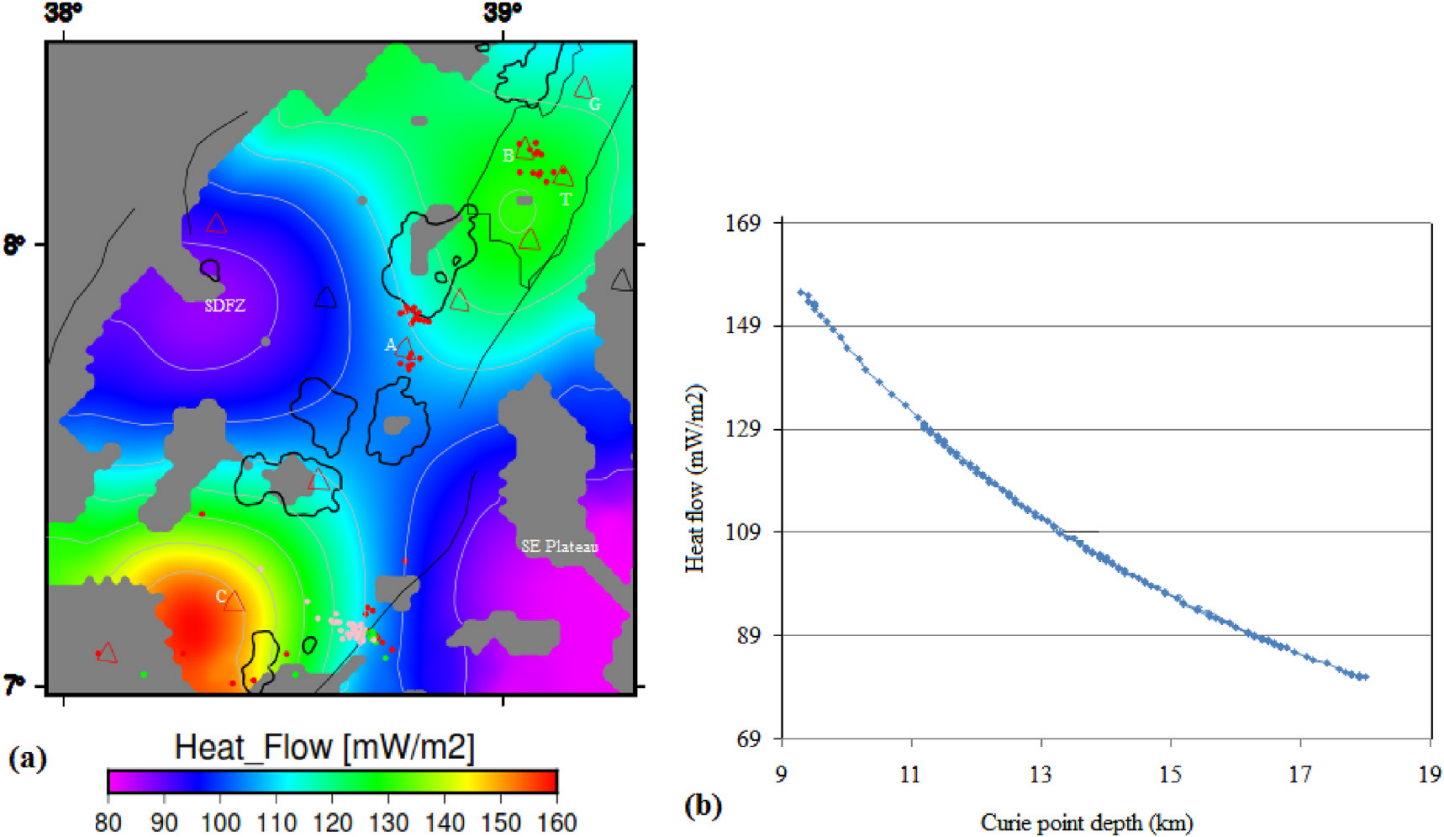
(a) Heat flow map of the central MER estimated from Curie point depths in Figure 7. (b) Curie point depth against heat flow curve of the central main Ethiopian rift reflects an inverse correlation between them. Red, green and pink circles represent the seismicity centers with depth less than 16 km, between 16 and 25 km and greater than 25 km, respectively. G, B, T, A and C stands for the Gedemssa, Bora-Bericcio, Tullu-Moye, Aluto and Correbetti central volcanoes. The acronyms include SDFZ and SE plateau stands for the Silti Debrezit fault zone and southeastern plateau, respectively.

In our study area, the estimated geothermal gradient values fluctuate between 32.4 and 65 °C/km (Figure 9a). Geothermal gradient values as low as 32.4 °C/km are found to occur beneath the SDFZ and southeastern plateau (Figure 9a). These low geothermal gradient values could be related with crustal thickening (Kassa et al., 2021) or due to large sedimentary cover (Korme et al., 2004) occurring beneath the southeastern plateau and the SDFZ. A high geothermal gradient occurs beneath the volcanic complexes including the Tulu Moye, Corbetti and Aluto volcanoes (Figure 9a). Beneath the Tulu Moye and Corbetti volcanoes, magnitude of the geothermal gradient reaches up to 65 °C/km, whereas, beneath the Aluto volcano it reaches up to ∼48 °C/km (Figure 10a). These results indicate that the volcanic complexes could be associated with high geothermal activity including hot springs, steam vents and fumarolic activities that hint an ongoing active magmatic process beneath the volcanoes.

Figure 9b reveals that the estimated geothermal gradient in the CMER decreases with increasing CPD values. For example, when the CPD values rise above 13.1 km, the geothermal gradient values become less than 43.8 °C/km.

#### 4.1.2 Comparison of CPD with heat flow

Calculation of heat flow (q) is dependent on CPD (D) variation, and is given by Eq. (6)

(8) $$q=k\left(\frac{580{}^{\circ}C}{D}\right)$$

where *k* is thermal conductivity taken here to be 2.5 W/m °C (Springer, 1999).

The estimated heat flow values range from 80 to 160 mW/m^2^ (Figure 10a) with an average value of 110 mW/m^2^. These estimated values are consistent with the results of several authors (Kerimov et al., 1989; Badalyan, 2000) in the areas they studied. According to Jessop et al. (1976), the average heat flow value (110 mW/m^2^) is thought to indicate anomalous geothermal conditions. The estimated maximum heat flow value (160 mW/m^2^) (Figure 10a) is a characteristics of volcanically active regions. According to Sandiford et al. (1998), this heat flow value (160 mW/m^2^) is of interest in geothermal exploration studies. Beneath the Aluto geothermal field, the estimated heat flow value reaches to a maximum of ∼110 mW/m^2^, which is comparable to the estimated average heat flow value for the whole of the CMER. The estimated low heat flow value (80 mW/m^2^) occurs beneath the SDFZ and the southeastern plateau (Figure 10a).

Figure 10b shows the estimated heat flow values decrease with increasing CPD values. For example, when the CPD values are increase to values above 13.1 km, the heat flow decreases to values below the estimated average value (110 mW/m^2^) beneath the study area. These decreasing heat flow values appear to occur due to the influence of the volcanic activities occurring beneath the study area.

#### 4.1.3 Comparison of CPD with lithospheric structures

The crustal/lithospheric mantle characteristics considered here are the gravity Moho depth, LAB depth, lithospheric mantle thickness and Conrad depth.

**Figure 11:**
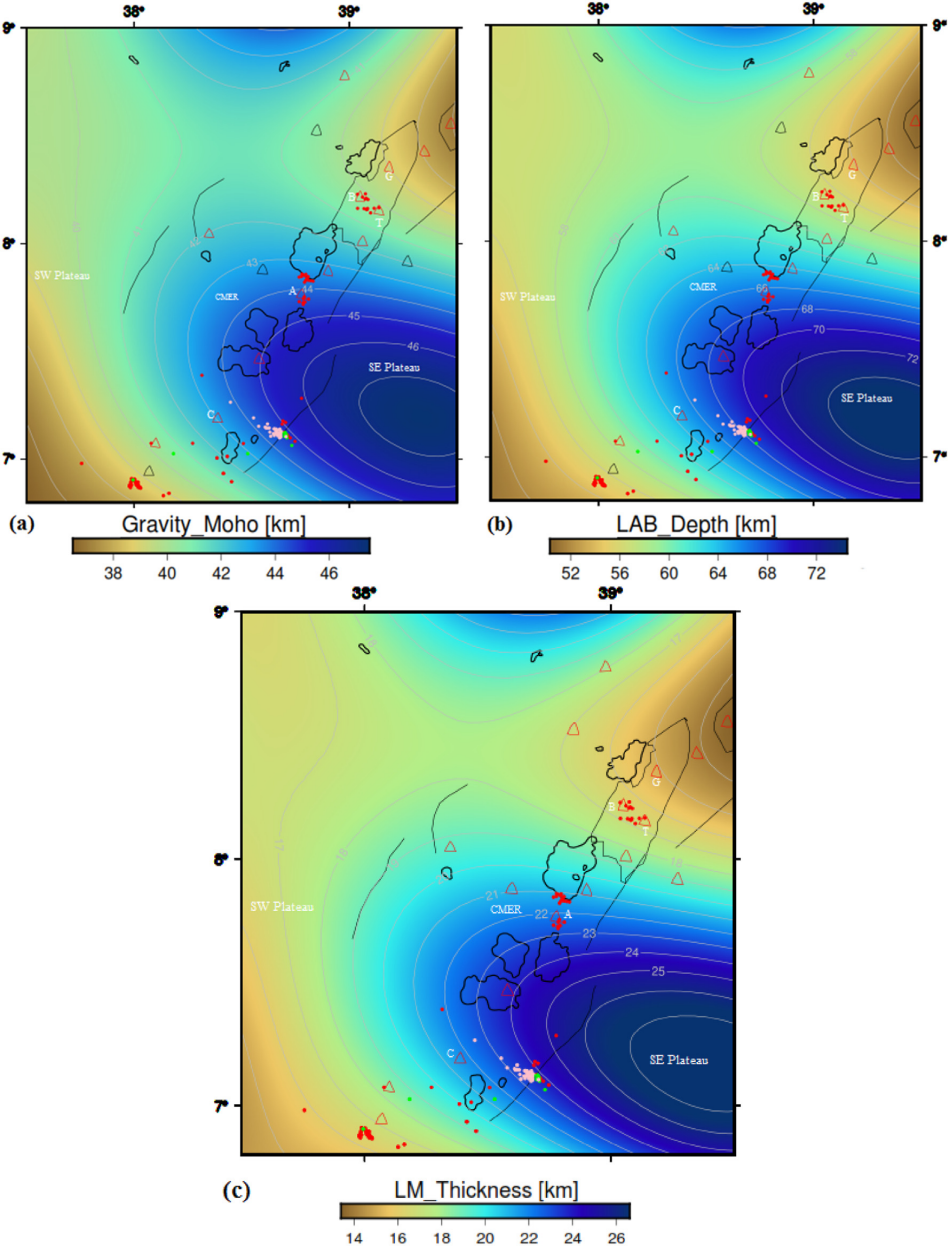
(a) Depth to Moho map of the central MER including the adjoining plateaus (from Kassa et al., 2021). (b) Lithosphere-asthenosphere boundary depth map of the central MER and surrounding regions obtained from our previous work (Kassa et al., 2022). Focal depth values, less than 16 km (red circles), greater than 25 km (pink circles) and between 16 and 25 km (green circles), obtained from previous published seismic works (Keir et al., 2006; Delvaux and Barth, 2010; Lapins et al., 2020; Ogden et al., 2021). Holocene (red triangles) and Pleistocene (black triangles) volcanoes are acquired from global volcanism programs **(**https://volcano.si.edu). BSF, G, BB, TM, A and C stands for the Gedemsa, Bora-Bericcio, Tulu-Moye, Aluto and Correbetti volcanic complex, respectively. The Acronym includes the SW Plateau, CMER and SE Plateau stands for the southwestern plateau, central Main Ethiopian rift and southeastern plateau, respectively.

The gravity Moho depth values computed by Kassa et al. (2021) using a Parker-Oldenburg inversion algorithm. The computed Moho depth (Figure 11a) varies from 36.5 to 47.47 km beneath the CMER and surrounding regions is determined to be comparable with the value determined using the Rayleigh wave/receiver function studies (Keranen et al., 2009). Our previous crustal thickness model results indicate that the Moho depth values fluctuate between 38 and ∼46 km beneath the central MER, between 36 and 42 km beneath the SW plateau and between 36 and 47.8 km beneath the SE plateau (Kassa et al., 2021). The Moho depth values determined using receiver function (Keranen et al., 2009) and gravity data (Kassa et al., 2021) have indicated occurrence of a relatively shallow crust beneath the northern MER as compared to the CMER.

The LAB depth values are computed by Kassa et al. (2022) using a Parker-Oldenburg inversion algorithm. The inversion results indicate that the LAB depth values vary between 52 and 72 km beneath the central MER, between 50 and 75.3 km beneath the SE plateau and between 50 and 65 km beneath the SW plateau (Figure 11b). Here, the computed LAB depth values are utilized to determine lithospheric mantle thickness estimated by subtracting the crustal thickness values (Kassa et al., 2021) from the inversion results of the LAB depth values (Kassa et al., 2022; Figure 11b). Thus, the estimated lithospheric mantle thickness values vary between 14 and ∼26 km beneath the central MER, between 14 and 27.5 km beneath the SE plateau and between 14 and 22 km beneath the SW plateau (Figure 11c).

**Figure 12:**
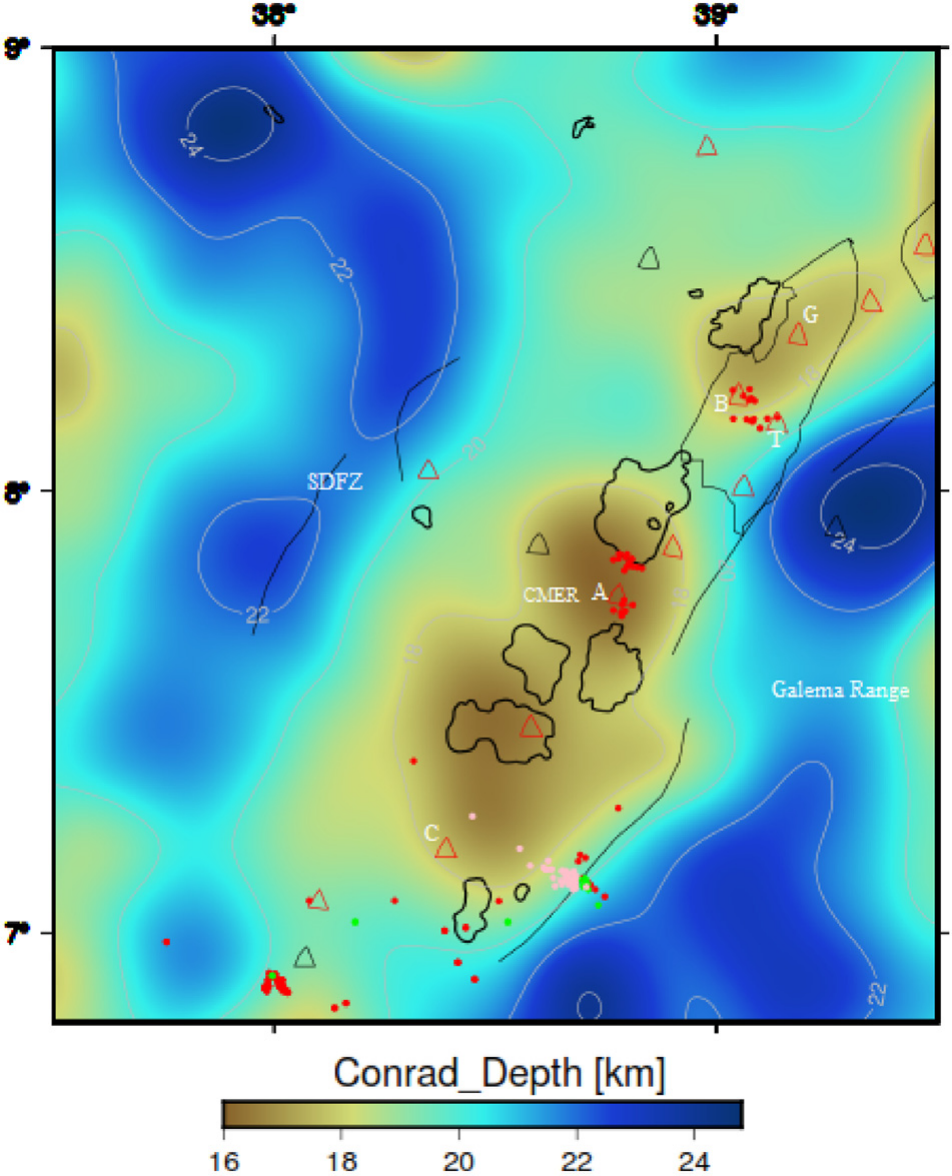
Conrad depth maps of the MER (a)/CMER (b) and its surroundings generated by using the Parker-Oldenburg method. G, B, T, A and C stands for the Gedemsa, Bora-Bericcio, Tulu-Moye, Aluto and Correbetti volcanic complex, respectively. Black solid lines, black and gray polygons represent the border faults, lakes and magmatic segments, respectively. SDFZ and CMER stands for the Silti Debrezit fault zone and the central main Ethiopian rift, respectively.

The Conrad depth considered here is generated by using the Parker-Oldenburg inversion algorithm (see section 3.2). The lowest (16 km) and highest (∼25 km) depths of the Conrad boundary are determined to occur beneath the central MER and southeastern/western plateaus, respectively (Figure 12). These estimated values are in agreement with the results of artificial seismic sounding experiments (Wang et al., 2002), who found that the depth to Conrad varies from 20 to 30 km in the Yunnan region. Figure 12, shows that the Conrad depth is relatively reduced to vary between 16 and 19.4 km beneath the central MER. These reduced Conrad depth values (16–19.4 km) are in agreement with the depth values (18–20 km) determined using wide angle controlled source seismic profiling (Maguire et al., 2006). In the central MER, the estimated shallowest Conrad depth value is 16 km. This value is comparable with the result of Muluneh et al. (2018), who estimated the boundary depth value (16 km) occurs between the brittle and ductile crust beneath the MER including our investigated region.

The depth to Conrad map (Figure 12) is thought to reveal the first order geomorphologic features of the CMER and surrounding regions. The morphologically low (16–19.4 km) trend of the depth to Conrad map (Figure 12) coincides with the trend of the observed maximum gravity anomaly (Figure 5b), magmatic segments, volcanic and seismicity centers in the CMER floor. Whereas, the morphological high values (19.5–∼ 25 km) of the depth to Conrad (Figure 12) beneath the southwestern and southeastern plateaus correlate with the gravity anomaly minima occurring beneath the flanks of the rift including the Fonko-Guraghe border fault (FGBF), the Galema Range and the Arboye border fault (ABF). Generally, our Conrad depth model outlined the low morphology of the rift depression and the high morphology of the rift escarpments (i.e., the FGBF, ABF and Galema Range).

##### 4.1.3.1 Comparison of Curie point depth with gravity Moho depth

To compare the estimated results of the CPD with the computed gravity Moho, we use the Moho depth values computed by Kassa et al. (2021) using a Parker-Oldenburg inversion algorithm.

**Figure 13:**
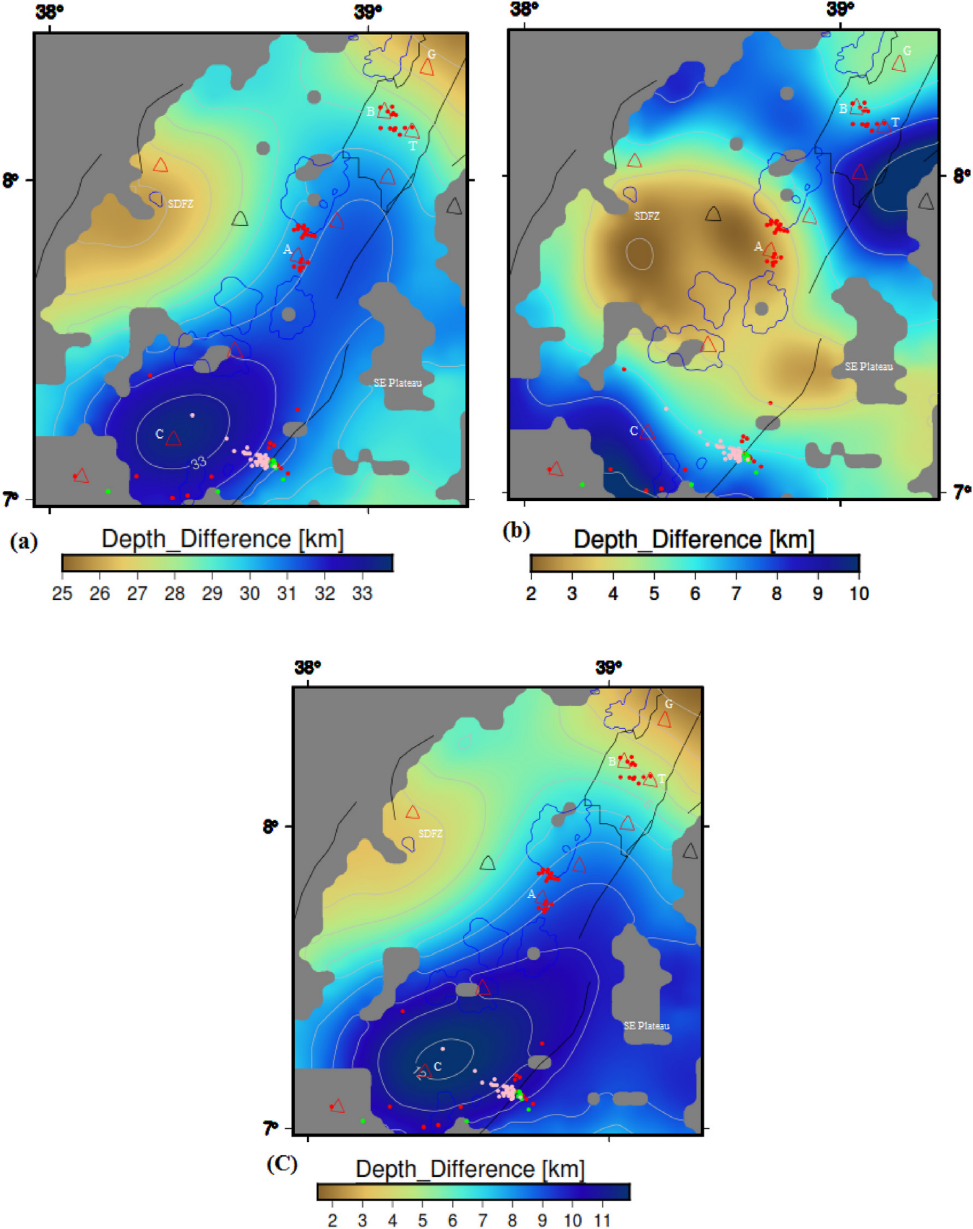
Depth difference between the gravity Moho and CPD (a), depth difference between the Conrad depth and CPD (b) and depth difference between the lithospheric mantle thickness and CPD (c) maps of the study area. Red, green and pink circles represent the seismicity centers with depth less than 16 km, between 16 and 25 km and greater than 25 km, respectively. The texts include G, B, T, A and C stands for the Gedemssa, Bora-Bericcio, Tullu-Moye, Aluto and Correbetti central volcanoes. Black solid lines, blue and black polygons represent the border faults, lakes and magmatic segment, respectively. The acronyms include SDFZ and SE plateau stands for the Silti Debrezit fault zone and southeastern plateau, respectively.

Comparing the CPD (Figure 7) with the gravity Moho depth (Figure 11a), one can judge that the CPD is less than that of the Moho depth. The difference between the Moho depth and CPD is depicted in Figure 13a. Figure 13a reveals that the minimum difference between the Moho depth and the CPD is ∼25.3 km beneath the SDFZ and Gedemssa volcano, and the maximum difference between them reaches up to ∼34 km beneath the Corbetti volcano. These results indicate that the difference between the Moho depth and CPD values fluctuate between ∼25.3 and ∼34 km. These fluctuations are determined to be consistent with the results of Speranza et al. (2016), who found that the difference between the Moho depth and CPD values range from 20 to 40 km beneath the Alps. In addition to this, Arnaiz-Rodríguez and Orihuela (2013) have shown that the Moho depth is larger than the CPD in marine area, whereas in continental areas either the CPD is smaller than the Moho depth or both show consistent variation between them.

More than 200 focal depth values (Keir et al., 2006; Delvaux and Barth, 2010; Lapins et al., 2020; Ogden et al., 2021) are employed to show correlation of the focal depth values with the CPD (Figure 7) and Moho depth values (Figure 11a) beneath the MER including the CMER.

The utilized focal depth values vary from 0.1 to ∼39 km, and are all less than the Moho depth values (Figure 11a). The estimated shallow CPD values (7.68–13.1 km) coincide with the utilized focal depth values beneath the CMER (Figure 7).

##### 4.1.3.2 Comparison of CPD with Conrad depth

The CPD can be defined as the depth where magnetized bodies lose their magnetic properties. The Conrad depth also defined as the bottom boundary of the lower crystalline basement layer. By comparing the CPD (Figure 7) with the Conrad depth (Figure 12), one can observe that the CPD occurs close to the Conrad depth. Differences between the Conrad depth and CPD are depicted in Figure 13b. The map reveals that the differences between the two depths range from ∼2 to 10 km depth. Wang et al. (2002) suggested that the CPD is an undulating surface and is located close to the Conrad discontinuity. Based on the results obtained by Wang et al. (2002) and the results of Figure 13b, we can argue that our estimated CPD and Conrad depth values fairly agree to each other.

#### 4.1.4 Comparison of CPD with lithospheric mantle thickness

Differences between the lithospheric mantle thickness and CPD are depicted in Figure 13c. The map (Figure 13c) reveals that the differences between the two depths range from ∼1.5 to 12.1 km depth. The discrepancy map (Figure 13c) indicates that in most of the study area except the Correbetti volcanic area the CPD occurs close to the lithospheric mantle thickness.

#### 4.1.5 Comparison of CPD with seismic wave velocity ratio anomalies

To correlate the estimated results of the CPD with earlier published seismic wave velocity ratio anomalies, we review the wave velocity ratio results determined by Dugda et al. (2005), Stuart et al. (2006), Keranen et al. (2009) and Cornwell et al. (2010).

**Figure 14:**
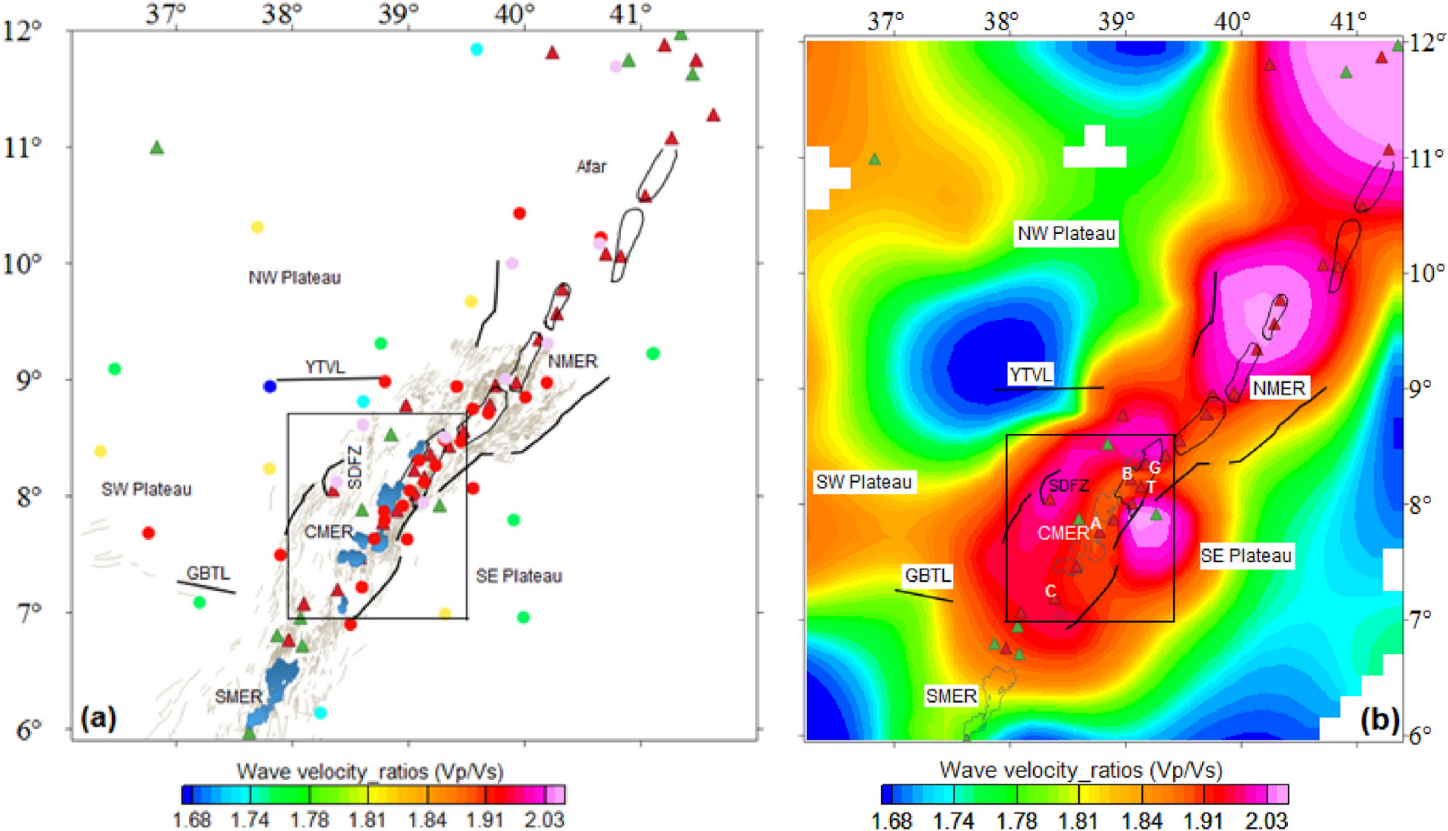
Distribution of the wave velocity ratio (a) and wave velocity ratio anomalies map (b) of the Ethiopian rift and surrounding plateaus (from Dugda et al., 2005; Stuart et al., 2006). The open black box shows the location of the study area. G, B, T, A and C stands for the Gedemssa, Bora-Bericcio, Tullu-Moye, Aluto and Correbetti central volcanoes. Black solid lines, blue and black polygons represent the border faults, lakes and magmatic segment, respectively. The acronyms include SDFZ, SE plateau, GBTL, SW plateau, YTVL, NW Plateau, SMER, CMER and NMER stands for the Silti Debrezit fault zone, southeastern plateau, Goba-Bonga tectonic lineament, southwestern plateau, Yerer-Tullu Wellel volcanotectonic lineament, northwestern plateau, southern, central and northern main Ethiopian rift, respectively.

The seismic wave velocity ratio values computed by Dugda et al. (2005) and Stuart et al. (2006) for the Ethiopian rift system/surrounding regions which incorporate the study area is depicted in Figure 14a, b. The Figure reveals the plotted and mapped seismic wave velocity ratio values vary in the range of 1.66–2.26 with an average value of 1.89 beneath the Ethiopian rift system including the surrounding plateaus. The figure also illustrates that the wave velocity ratio vary in the range of 1.83–2.26 with an average is 2.05 beneath the floor of the CMER (Figure 14a, b). Keranen et al. (2009) shown that the lower crustal wave velocity ratio values fluctuate between 1.81 and 1.88 in the Ethiopian rift system. Cornwell et al. (2010) determined that the mean crustal wave velocity ratio values vary between 1.84 and 1.92 beneath the NMER.

Comparing the CPD map (Figure 7) with the crustal wave velocity ratio anomaly map (Figure 14b), one can observe that the relatively high wave velocity ratio values correlate with the shallow CPD beneath volcanic areas including the Corbetti, Aluto, Tullu-Moye, Bora Bericcio and Gedemssa. Xu et al. (2005) and Li et al. (2009) gave the following probable explanation of about the relatively high wave velocity ratios observed in the areas they studied. Xu et al. (2005) explained the relative high wave velocity ratio indicates that the lower crust material composition is enhanced by the magnesium iron content. Li et al. (2009) explained the relatively high wave velocity ratio is a consequence of the occurrence of lower crustal partial melt because of elevated temperature at the base of the lower crust.

Following the explanations given by Xu et al. (2005) and Li et al. (2009), Figure 7 indicates that the SDFZ has a deep CPD which is in line with the elevated wave velocity ratio anomalies inferred by Dugda et al. (2005) and Stuart et al. (2006) (Figure 14b) and low crustal wave velocity anomaly determined by Chambers et al. (2019).

### 4.2 Joint 2D magnetic and gravity forward modelling

**Figure 15:**
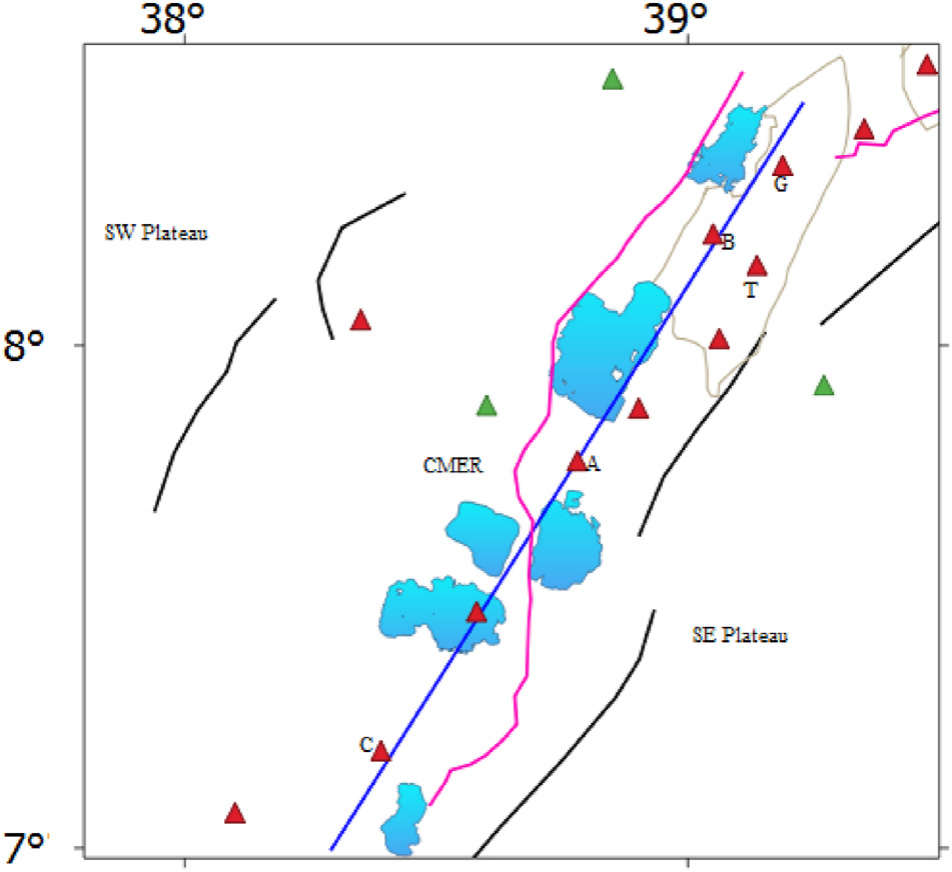
Profile lines include seismic profile line (Pink line) from previously published work of Maguire et al. (2006) and selected magnetic/gravity profile line (blue line) of the study area. The texts include G, B, T, A and C stands for the Gedemsa, Bora-Bericcio, Tullu-Moye, Aluto and Corbetti volcanic areas. The Central Main Ethiopian Rift (CMER) comprises different rift lakes (Blue polygons) and it is surrounded by the southwestern plateau (SW plateau) to the south-west and Southeastern plateau (SE plateau) to the south-east. Red and green triangles represent the Holocene and Pleistocene volcanoes, respectively.

**Figure 16:**
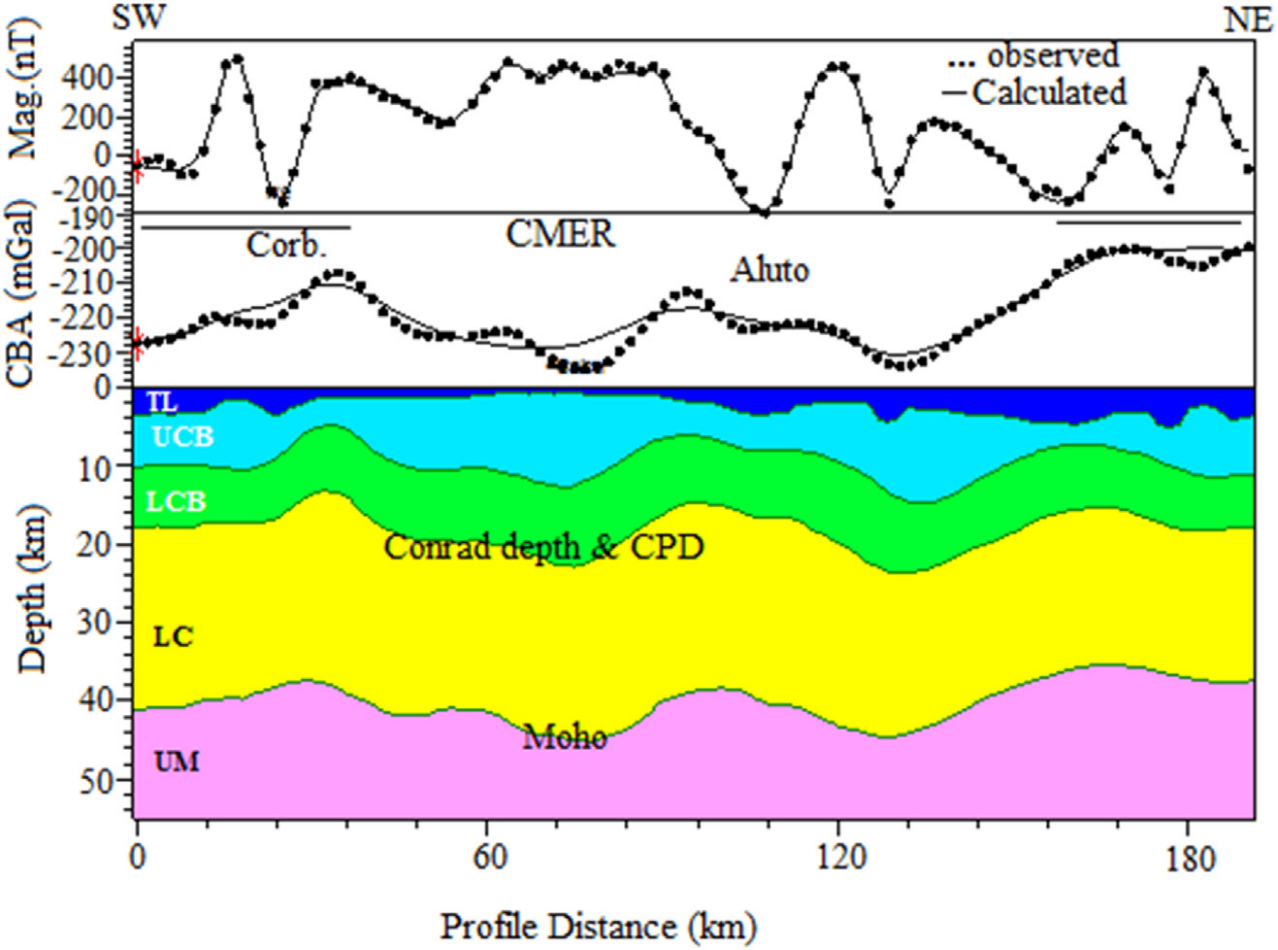
Two dimensional (2D) joint magnetic and gravity model of the study area, which is running along the study area. The abbreviations comprising the TL, UCB, LCB, LC and UM are stands for the top layer, upper crystalline basement, lower crystalline basement, lower crust and upper mantle.

Joint 2D Magnetic and gravity forward modelling considered here is essential to improve the crustal structure of the area of interest rather than individually generated gravity or magnetic modelling. Our joint model is constrained by apriori data obtained from different sources (e.g., Alemu, 1992; Mammo, 2010; Maguire et al., 2006; Blaikie et al., 2014; Kassa et al., 2021, 2022, Table 2) and it is calculated using the CBA/pole reduced magnetic anomaly profile as shown in Figure 15. Table 2 shows the subsurface characteristics include the depth variations of the bottom interface of the subsurface layers generated from 3D gravity structural inversion (Kassa et al., 2021, 2022), susceptibility and density values obtained from different sources (e.g., Alemu, 1992; Mammo, 2010; Maguire et al., 2006; Blaikie et al., 2014). These subsurface characteristics in this work utilized as preliminary information in order to construct joint 2D magnetic and gravity forward modelling. Here, we argue that the CPD is fairly equivalent with the Conrad depth (detail of the comparison between the CPD and Conrad depth is shown in sub-section 4.1.3.2). Our joint 2D magnetic and gravity model along the study area with the preferred profile (Figure 15) provides a fairly fit between the calculated and observed magnetic and gravity anomalies with an error of 28.961 nT and 2.98 mGal respectively (Figure 16). All existing literature states that the areas that have geothermal resources, tectonically, volcanically and seismically active regions are characterized by shallow CPD and crustal thickness (e.g., Tessema and Antoine, 2004; Woldetinsae, 2005; Maguire et al., 2006; Saibi et al., 2015; Elbarbary et al., 2022). This scenario is very well depicted in our 2D joint modelling. Arnaiz-Rodríguez and Orihuela (2013) have indicated that in continental areas either the CPD is smaller than the Moho depth or both show consistent variation between them. This scenario is very well depicted in our 2D joint modelling (Figure 16) indicates that the CPD is smaller than the Moho depth along the study area with the preferred profile (Figure 15).

**Table 2:** Subsurface characteristics include the bottom interface of the subsurface layers (Kassa et al., 2021, 2022), susceptibility and density values acquired from different sources (e.g., Alemu, 1992, Mammo, 2010; Maguire et al., 2006; Blaikie et al., 2014).

Layers	Depth variations of the bottom interface of the subsurface layers (km)	Density (g/cc)	Susceptibility	References
Top layer	1.5–7.045	**2.6**	0.0001825–0.016	Alemu, 1992; Mammo (2010); Maguire et al. (2006); Blaikie et al. (2014); Kassa et al. (2022)
Upper crystalline basement	5–15	2.7	0.000795	Maguire et al. (2006); Blaikie et al. (2014); Kassa et al. (2022)
Lower Crystalline basement	16–25	2.79	0.0033	Maguire et al. (2006); Mammo (2010); Kassa et al. (2022)
Lower Crust	38- ∼ 46	2.94	0	Maguire et al. (2006); Kassa et al. (2021)
Upper Mantle	Up to 60	3.27	0	Maguire et al. (2006); Kassa et al. (2022)

## 5 Conclusion

This study is dedicated to calculation of Conrad and Curie point depth in relation with heat flow and Moho undulation beneath the central MER and surrounding regions. The Conrad depth is estimated based on gravity data inversion. The CPD is calculated based on Fourier domain RTP magnetic anomaly data. The Fourier domain RTP magnetic anomaly data are divided into 15 sub-window areas, and then for each sub-window areas of spectral analysis is employed to calculate the centroid, top and CPD. Our results showed that the depth to the top of the magnetic source values range from 1.75 to 5.97 km. The estimated depth values of the centroid vary from 5.04 to 11.4 km in the CMER. The inferred CPD values fluctuate between 7.68 and 20.3 km. The Conrad depth model considered in this study to show an increase Conrad depth values from the CMER (16 km) to both sides of the plateaus (∼25 km).

Comparison of the Moho depth and CPD in the region shows that the CPD is smaller than that of the Moho depth. On the contrary, comparison of the Conrad depth and the CPD in the study area shows that the CPD values seem to be close to the Conrad depth values.

The Heat flow and geothermal gradient beneath the CMER are calculated based on the estimated CPD. The estimated CPD values appear to be inversely correlated with the heat flow and geothermal gradient values determined for the study area. The volcanic complexes including the Tulu Moye, Gedemssa, Aluto and Corbetti are characterized by low CPD values (up to 7.68–13.1 km) and high heat flow values (up to 110–160 mW/m^2^), being associated with high wave velocity ratio and relatively thin crust. The southeastern plateau and the SDFZ are characterized by high CPD values (reaches up to 20.3 km) and low heat flow values (up to 80–91.9 mW/m^2^), in agreement with elevated wave velocity ratio values determined by several authors in the region.

## Declarations

### Author contribution statement

Muluken Kassa: Conceived and designed the experiments; Performed the experiments; Analyzed and interpreted the data; Contributed reagents, materials, analysis tools or data; Wrote the paper.

Abera Alemu: Analyzed and interpreted the data; Wrote the paper.

Ameha Muluneh: Contributed reagents, materials, analysis tools or data; Wrote the paper.

### Funding statement

This research did not receive any specific grant from funding agencies in the public, commercial, or not-for-profit sectors.

### Data availability statement

Data will be made available on request.

### Declaration of interest's statement

The authors declare no conflict of interest.

### Additional information

No additional information is available for this paper.
